# JAK1 Is a Novel Target of Tumor- and Invasion-Suppressive microRNA 494-5p in Colorectal Cancer

**DOI:** 10.3390/cancers16010024

**Published:** 2023-12-20

**Authors:** Nitin Patil, Omar G. Abdelrahim, Jörg H. Leupold, Heike Allgayer

**Affiliations:** Department of Experimental Surgery-Cancer Metastasis, Mannheim Medical Faculty, Ruprecht-Karls University of Heidelberg, 69047 Heidelberg, Germany; nitin.patil@medma.uni-heidelberg.de (N.P.); omar.abdelrahim@medma.uni-heidelberg.de (O.G.A.); joerg.leupold@medma.uni-heidelberg.de (J.H.L.)

**Keywords:** miR-494-5p, miRNAs, colorectal cancer, JAK1, invasion, migration

## Abstract

**Simple Summary:**

MicroRNA (miR) 494-5p has been associated with cancer progression, but molecules mediating this are not well understood. Here, we show that the 3’UTR of JAK1 is physically targeted, and JAK1 is downregulated in expression by miR-494-5p in colorectal cancer (CRC). Additionally, CRC cell proliferation, spontaneous and IL-4-induced invasion, and migration were significantly reduced by this miR, as well as IL-4-induced phosphorylation of JAK1, STAT6, and AKT. High JAK1 expression significantly correlated with reduced patient survival. Together, our findings suggest JAK1 as a novel target of miR-494-5p, with its translational repression contributing to CRC progression and the initial steps of metastasis.

**Abstract:**

MiR-494-5p expression has been suggested to be associated with colorectal cancer (CRC) and its metastases in our previous studies. However, functional investigations on the molecule-mediating actions of this miR in CRC are lacking. In silico analysis in the present study revealed a putative binding sequence within the 3′UTR of JAK1. Overexpression of miR-494-5p in cultured CRC significantly reduced the luciferase activity of a reporter plasmid containing the wild-type JAK1-3′UTR, which was abolished by seed sequence mutation. Furthermore, the overexpression of miR-494-5p in CRC cell lines led to a significant reduction in JAK1 expression, proliferation, in vitro migration, and invasion. These effects were abolished by co-transfection with a specific double-stranded RNA that inhibits endogenous miR-494-5p. Moreover, IL-4-induced migration, invasion, and phosphorylation of JAK1, STAT6, and AKT proteins were reduced after an overexpression of this miR, suggesting that this miR affects one of the most essential pathways in CRC. A Kaplan–Meier plotter analysis revealed that patients with high JAK1 expression show reduced survival. Together, these data suggest that miR-494-5p physically inhibits the expression of JAK1 at the translational level as well as in migration and invasion, supporting the hypothesis of miR-494-5p as an early tumor suppressor and inhibitor of early steps of metastasis in CRC.

## 1. Introduction

CRC is one of the leading causes of cancer-related death, mainly due to advanced progression [1]. Recent epidemiological data suggest that the incidence and mortality of CRC have slightly declined, although there is a rising concern regarding metastasis, especially in the case of young adults [2]. The development of CRC is a highly complex process involving several molecules and cellular pathways that contribute to CRC carcinogenesis from benign adenomas. Amongst other processes, genetic alterations, such as a mutation-derived activation of oncogenes and inactivation of tumor suppressor genes, have been shown to be key players that drive tumor development and the further advancement of cancer [3,4]. Several publications, including ours [5,6,7,8,9], have demonstrated that miR expression is deregulated in various cancers, including CRC, leading to abnormal translational regulation of target genes, thus affecting vital biological processes [10,11,12]. Consequently, during the last decade, miRs have been established, at least in part, as oncogenes or tumor suppressors, and they have become an indispensable part of the ‘hallmarks of cancer’ [13,14,15,16]. The mechanisms affecting miR dysregulation include, amongst others, an amplification or deletion of miR genes [17], epigenetic deregulation [18], transcriptional regulation of miR genes [6,19], or deregulations within miR biogenesis pathways [13,16,20,21].

Our group has already investigated several miRs in the past that play a crucial role in CRC development or metastasis, in part employing its own data sets based on a systematic expression analysis of human-resected tumor tissues for hypothesis generation [8,17,22]. Following this strategy, we have identified several key miRs deregulated in CRC tumor samples compared with the corresponding normal tissue counterparts [8]. For the present study, we have shortlisted miR-494-5p as a potentially interesting candidate molecular player in CRC since it has been demonstrated that an abnormal expression of this miR is crucial for the development of several cancer types, although the list of interesting target molecules regulated by this miR that are of high interest for cancer development and progression still needs to be extended [23,24,25,26,27,28,29,30,31,32,33,34].

JAK1 is part of the JAK/STAT signaling pathway and is known as one of the central communication pathways for (cancer) cell function controlled by various cytokines and growth factors [35]. JAKs are commonly associated with cytokine receptors for the transmission of signals responsible for the recruitment of one or more STAT proteins [35]. Tyrosine-phosphorylated STATs dimerize and are transferred into the nucleus to regulate specific genes. The same applies to JAK1, which acts as a key player for the JAK/STAT signaling pathway in the development and progression of several cancers [36,37,38,39]. Specifically, cytokines like IL-4 and IL-13 use JAK1 to initiate cellular signaling through STAT6, which is a transcription factor required for many of their biological functions. Moreover, it has already been shown that this signaling pathway is highly activated in tumors to promote, e.g., tumor metastasis in CRC and melanoma [40]. Therefore, it is not surprising that the deregulation of JAK/STAT proteins is mainly associated with poor clinical prognosis and/or reduced overall survival in patients with solid tumors and hematological malignancies [41].

In our present work, we identified JAK1 as a target of miR-494-5p. Over and above in silico tools and pathway analysis, we demonstrate the functional presence of an miR-494-5p physical binding site within the 3′UTR of JAK1 and show that miR-494-5p overexpression in human CRC cells significantly inhibits JAK1 3′UTR activity and JAK 1 expression at the mRNA and protein level. Finally, we show that the translational targeting of JAK1 by miR-494-5p inhibits cancer cell migration and invasion, thereby supporting a tumor-suppressive role of miR-494-5p in the initial steps of the metastatic cascade in CRC.

## 2. Materials and Methods

### 2.1. Cell Lines

Human cell lines used in this study (COLO320, RKO, HCT116, SW480, SW620, Geo, CaCo2, WiDr, HCT15, and HT29) were obtained from ATCC or The Leibniz Institute DSMZ-German Collection of Microorganisms and Cell Cultures GmbH (Germany). All cell lines were maintained in the medium recommended by ATCC in a humidified atmosphere with 5% CO_2_ at 37 °C. As soon as they reached a confluence above 70%, cells were washed with PBS, trypsinized, and further cultivated.

### 2.2. Bioinformatics Analysis and Target Identification

The following online databases were used to discover potential target molecules: miRWalk (http://mirwalk.umm.uni-heidelberg.de/, last accessed on 27 April 2022), miRDB (http://mirdb.org/mirdb/index.html, last accessed on 27 April 2022), and TargetScan Release 8.0 (https://www.targetscan.org/vert_80/, last accessed on 27 April 2022). Only genes containing sequences for miRs that were common in all three databases and exceeded a high probability of binding were selected. These common gene signatures were then imported into the DAVID online tool platform (https://david.ncifcrf.gov/, last accessed on 28 April 2022), and a functional KEGG pathway analysis was carried out subsequently. Only the most significant pathways related to cancer were considered for final analysis. The resulting data were then exported to Excel sheets based on their frequency, and a graph of -log10 (*p*-value) was plotted for visualization.

Post-progression survival (PPS), recurrence-free/relapse-free survival (RFS), and overall survival (OS) depending on JAK1 in CRC tissues, together with Mantel–Cox log-rank tests, were evaluated using default settings of the Kaplan–Meier KM plotter website (https://kmplot.com/analysis/index.php?p=background, last accessed 10 September 2023) [42].

### 2.3. Total RNA Isolation from Cell Lines and cDNA Synthesis

The isolation of total RNA from cell lines was carried out using the RNeasy Kit (QIAGEN GmbH, 40724 Hilden, Germany; catalog number 74104) or TRIzol reagent (Thermo Fisher Scientific, 63303 Dreieich, Germany; Catalog number: 15596026). All protocols were applied in accordance with the manufacturer’s recommendation. The concentrations of total RNA and DNA were quantified using the NanoDrop spectrophotometer (Thermo Fisher Scientific, 63303 Dreieich, Germany). First-strand cDNA synthesis was carried out using the miScript II RT Kit (QIAGEN GmbH, 40724 Hilden, Germany; catalog number 218160). Equal amounts of total RNA were reversely transcribed in a 20 µL reaction volume at 37 °C for 60 min, followed by an incubation of 5 min at 95 °C to inactivate the enzyme.

### 2.4. Genomic DNA Isolation and Plasmid Isolation

The genomic DNA was isolated using the QIAamp DNA Mini Kit (QIAGEN GmbH, 40724 Hilden, Germany; catalog number 51304). Plasmid DNA was isolated using the QIAPprep Spin miniprep kit or QIAgen plasmid Maxi Kit (QIAGEN GmbH, 40724 Hilden, Germany; catalog numbers 27106 and 12163). All protocols were applied in accordance with the manufacturer’s recommendations.

### 2.5. Quantitative Real-Time PCR Primers, miRs, and si-RNAs

The quantitative real-time PCR was carried out using the Fast SYBR Green Master Mix (Thermo Fisher Scientific, 63303 Dreieich, Germany; catalog number 4385612) using the following primers: Hs_JAK1_1_SG QuantiTect Primer Assay (catalog number 249900; GeneGlobe ID QT00050225), Hs_ACTB_1_SG QuantiTect Primer Assay (catalog number 249900, GeneGlobe ID QT00095431), Hs_RNU6-2_11 miScript Primer Assay (catalog number 218300, GeneGlobe ID MS00033740), and Hs_miR-494-5p_1 miScript Primer Assay (catalog number 218300, GeneGlobe ID MS00045962) (QIAGEN GmbH, 40724 Hilden, Germany). The relative gene expression was measured by applying the 2^−ΔΔCt^ method. The miR or messenger RNA levels in the samples were normalized by RNU6 or β-actin against the respective target gene expression. The mirVana miR mimic for has-miR-494-5p (assay ID MC26467, catalog number 4464066), miRNA mimic negative control # 1 (catalog number 4464058), mirVana miR inhibitor fhashsa-miR-494-5p (assay ID MH26467, catalog number 4464084), miR inhibitor negative control #1 (catalog number AM17010), si-RNA silencer for JAK1 (assay ID 217, Catalog number AM51331), and silencer negative control # 1 (catalog number AM4635) were purchased from Thermo Fisher Scientific, 63303 Dreieich, Germany. MirVana™ miRNA mimics are made to imitate endogenously expressed mature miRs. In contrast to mirVana™ miRNA inhibitors, they are double-stranded. MirVana™ miRNA inhibitors are made to bind and inhibit endogenously expressed miRs. Negative Controls and mirVana™ miRNA Inhibitor Negative Controls are designed to minimize any unspecific side effects in the experimental setup. Therefore, both are designed and pre-tested to prevent any unwanted targeting of any human, rat, or mouse genes.

### 2.6. Plasmid Generation and Site-Directed Mutagenesis

Primers were designed, flanking the putative miR-494-5p binding site as mentioned below, to obtain a fragment size of 681 base pairs to amplify the 3′UTR region of the JAK1 (NCBI gene ID 3716) (forward primer 5′-GTAGCTAGCCCTTCTCCTGCAACAAATGC 3′ (NheI overhang), reverse primer 5′-AAACCTAGGAGCACTGGCACAGGCTTAGT 3′ (AvrII overhang)). The PCR reaction was performed using the protocol provided with Phusion High-Fidelity DNA Polymerase (Thermo Fisher Scientific, 63303 Dreieich, Germany; catalog number F530L). The resulting amplified PCR product was further purified using the QIAquick PCR purification kit (QIAGEN GmbH, 40724 Hilden, Germany; catalog number 28106) and restriction digested (NheI and AvrII). In parallel, the pLightSwitch reporter 3′UTR plasmid (SwitchGear Genomics, 1310 La Hulpe, Belgium) was digested with the same restriction enzymes and was SAP treated and ligated together with a purified PCR fragment. The obtained construct was further verified using Sanger sequencing employing sequencing primers from the plasmid vector as recommended (3′UTR insert sequencing primers) by the provider (SwitchGear Genomics, 1310 La Hulpe, Belgium). For mutation of the cloned JAK1 3′-UTR seed sequence, the QuickChange II XL Site-Directed Mutagenesis Kit (Agilent, 69123 Heidelberg, Germany catalog number 200517) was used. The reactions were carried out in line with the manufacturer’s protocol, and the amplified plasmid was digested with DpnI. The following primers were used for mutation of the miR-494-5p binding site: forward primer 5′-TTCCACAAGGAGGAGTGCTTAAGTGCTTTCAAATATTCCGGAATTCCGTATGTGTCACTAAGTTACTGGTACCAAATTTAAAGAG-3′ and reverse primer 5′-CTCTTTAAATTTGGTACCAGTAACTTAGTGACACATACGGAATTCCGGAATATTTGAAAGCACTTAAGCACTCCTCCTTGTGGAA-3′. The mutated sequence was further verified using Sanger sequencing, using the recommended standard primers.

### 2.7. Transfection and Stimulation of Cultured Human CRC Cells

All transfection experiments were in line with the protocol recommended for Lipofectamin 2000 or Lipofectamine 3000 Reagent (Thermo Fisher Scientific, 63303 Dreieich, Germany; catalog number 11668027 and catalog number L3000008, respectively). Before transfection, cells were washed with PBS and seeded overnight with Opti-MEM reduced serum media (Thermo Fisher Scientific, 63303 Dreieich, Germany; catalog number 11058021). The transfection media were replaced after 4 to 6 h with the appropriate complete medium and incubated further at 37 °C as required.

Stimulation of cells was carried out as described in [43]. Briefly, 5 × 10^5^ cells were seeded, transfected, and 24 h later, starved overnight with 1 mL of serum-free medium. Subsequently, the cells were stimulated with IL-4 (rh IL-4, ImunoTools GmbH, 26169 Frisoythe, Germany; catalog number 11340043) for the indicated time points at 37 °C. Immediately after stimulation, total protein was isolated as described below in Section 2.12.

### 2.8. Dual-Luciferase Reporter Assays

Cells were transfected with wild-type or mutant JAK1 3′UTR plasmid, together with either hsa-miR-494-5p mimic (assay ID MC26467; catalog number 4464066) or hsa-miRNA mimic negative control (catalog number 4464058) (Thermo Fisher Scientific, 63303 Dreieich, Germany) using Lipofectamin 2000 (Thermo Fisher Scientific, Germany). Forty-eight hours post-transfection, the luciferase activity was measured using a dual-luciferase reporter assay system (Promega Corporation, 69190 Walldorf, Germany; catalog number E1960).

### 2.9. Proliferation Assay

For the proliferation measurement, cells (1 × 10^4^) were seeded in a clear 96-well plate. After 24 h, the cells were transfected individually with hsa-miR-494-5p (mimic), hsa-miRNA negative control, hsa-miR-494-5p inhibitor, or hsa-miRNA inhibitor negative control, using Lipofectamin 2000. Measurements were performed at the indicated points in time using the CellTiter-Glo Luminescent cell viability assay kit (Promega Corporation, 69190 Walldorf, Germany; catalog number G7570). A graph of time point versus absorbance at 490 nm was plotted with ±SD of the mean.

### 2.10. Migration Assays and Invasion Assays

For invasion assays, each transwell chamber (12 mm transwell, 0.4 µm membrane insert, Corning Inc., Glendale, USA; catalog number 3460) was pre-coated with matrigel (10 µg) using corning matrigel growth factor reduced basement membrane matrix, LDEV-free (Corning Inc., Glendale, USA; catalog number 354230) and air dried under sterile conditions in a laminar hood overnight. Transwell chambers without matrigel were used for migration assays.

Cells were transfected either with hsa-miR-494-5p mimic, hsa-miRNA negative control, hsa-miR-494-5p inhibitor, or hsa-miRNA negative control using the Lipofectamin 2000. Twenty-four hours after transfection, cells were washed with PBS, trypsinized, and re-suspended in PBS. Subsequently, cell suspensions containing 1 × 10^5^ cells for invasion assay or 5 × 10^4^ cells for migration assay were prepared in 100 µL serum-free medium containing BSA (0.1%) and inoculated on the upper chamber of the wells. Supplemented cell culture medium (500 µL) was included in the lower chamber as a chemoattractant. Twenty-four hours after incubation, cells were trypsinized (top and bottom of the transmembrane), washed with PBS, and transferred to white 96-well plates for measurement. The CellTiter-Glo luminescent cell viability assay kit (Promega Corporation GmbH, 69190 Walldorf, Germany; catalog number G7570) was used to measure migrated or invaded cells.

### 2.11. Immunoblot (Western Blot) Analysis

The cell lysis was performed essentially as described by Dikic and colleagues [44]. Protein quantification of lysates was routinely performed by a colorimetric assay Pierce BCA Protein Assay Kit (Thermo Fisher Scientific, Germany; catalog number 23225). Equal amounts of total protein per sample were subjected to SDS-PAGE (10%) western blot gels and, after separation, transferred to nitrocellulose membranes using a semi-dry blotter. Membranes were blocked with 5% (*w*/*v*) non-fat dry milk dissolved in TBS containing 0.1% Tween-20 and incubated with primary antibodies in 2.5% (*w*/*v*) BSA in TBST at 4 °C overnight, followed by incubation with secondary antibodies conjugated to horseradish peroxidase diluted in TBST for 1 to 2 h at room temperature. For the detection of ß-Actin, NET-gelatin (0.15 M NaCl; 5 mM EDTA; 5 mM H_2_NC(CH_2_OH); 0.05% (*v*/*v*) Triton X-100; 0.25% (*w*/*v*) gelatin) was used for blocking membranes and dilution of antibody. Finally, membranes were incubated with Western Lightning Plus, Chemiluminescent Substrate (PerkinElmer, 63110 Rodgau, Germany; catalog number NEL103E001EA) for an appropriate time, and luminescence was detected with an imaging system. The following primary antibodies were obtained from Cell Signaling Technology, 2316 Leiden, The Netherlands and diluted as described: JAK1 (catalog number 3332, 1:1000), Phospho-JAK1 (Tyr1034/1035) (D7N4Z) (catalog number 74129, 1:1000), STAT6 (catalog number 9362, 1:1000), Phospho-STAT6 (Tyr641) (catalog number 9361, 1:1000), AKT (pan) (C67E7) (catalog number 4693, 1:1000), and Phospho-AKT (Ser473) (catalog number 9271, 1:1000). Anti-ß-Actin antibody AC-15 was obtained by Merck, Germany (catalog number A1978, 1:50.000). The following secondary antibodies were purchased from Jackson ImmunoResearch, Europe Ltd., Ely CB7 4EX, United Kingdom and diluted 1:50.000: Peroxidase AffiniPure Goat Anti-Rabbit IgG (H + L) (Code number 111-035-144) and Peroxidase AffiniPure Goat Anti-Mouse IgG (H + L) (Code number 115-035-146). Uncropped blots and densitometry values for all Western blots shown in this publication are shown in Supplementary Figures S1–S5.

### 2.12. Statistical Analysis

All experiments were carried out in two or three biological replicates, if not stated otherwise, and all values were considered for calculating the significance level. Statistical analysis was performed using the GraphPad Prism software 9.5.0 or using Microsoft Excel (microsoft office professional plus 2021). The Student’s *t*-test was used to compare differences between groups. In all cases, a *p-*value ns = *p* > 0.05, * = *p* ≤ 0.05, ** = *p* ≤ 0.01, and *** = *p* ≤ 0.001 is shown in figures unless otherwise stated, with ‘ns’ representing a value that is not significant. Data were represented as the mean ± standard deviation. For all survival analyses, the default settings of the KM plotter website were used [42]. The significance was computed using the Mantel–Cox log-rank test. *p* < 0.05 was defined as significant and *p* < 0.1 as a trend.

## 3. Results

### 3.1. JAK 1 Is an In Silico Target of miR-494-5p, and Associated with Poor Prognosis

**Figure 1:**
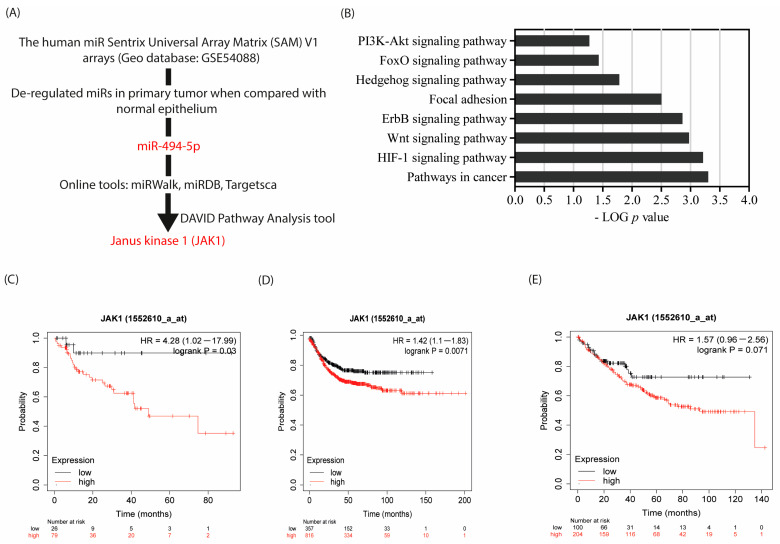
Identification of potential miR-494-5p targets and functional pathway analysis. (**A**) Schematic representation and workflow employed for miR identification, selection, and target prediction using TargetScanHuman_8.0, miRDB, and miRWalk online tools. (**B**) KEGG pathway analysis using DAVID tool shows the connection between our pool of 169 potential target genes with common cellular pathways. The bar corresponds to the log10 (*p*-value) of genes found in our pathway enrichment analysis. Survival plots based on JAK1 expression in CRC patients samples: (**C**) PPS, (**D**) RFS, and (**E**) OS provided by the KM plotter website, using default settings for the statistical analyses. Kaplan–Meier survival analysis and Mantel–Cox log-rank *p*-values are shown.

In one of our previous publications [8], we identified miR signatures exclusively deregulated in tumor tissues when compared with their corresponding normal counterparts. Based on this analysis, we decided to perform a functional analysis of miR-494-5p as the objective of the present study (Figure 1A). To identify potential direct translational targets of miR-494-5p, we combined three methods. In the first approach, we analyzed established public online databases specialized for miR-target prediction (TargetScanHuman_8.0, miRDB, and miRWalk). We obtained a pool of 169 potential target genes that were commonly identified for this miR within all of these databases, which was used to further streamline potential target identification (Supplementary Table S1). In the second approach, to achieve a rigorous pathway selection amongst potential miR target candidates, the DAVID analysis platform was employed. Amongst others, the PI3K-AKT signaling pathway, in particular, was predicted to be targeted by the translational repression exerted by miR-494-5p (Figure 1B). Therefore, we focused our present study on potential targets within, or related to, the PI3K-AKT signaling pathway. In line with promising wet lab investigations (see below), JAK1 came into focus as a miR target for our current investigations.

As a third line of support for our selection, the clinical impact of JAK1 was investigated using the KM plotter website [42]. The KM plotter has access to over 50,000 samples evaluated by gene expression arrays and RNA sequencing. The program is able to compute the impact of any gene of interest alone or in combination with other genes on patient survival in various tumor entities. Gene Expression data, relapse-free survival, and overall survival data are adopted from established databases, in particular, GEO, EGA, and TCGA. The database is handled by a PostgreSQL server, which integrates gene expression and clinical data simultaneously. Here, PPS and RFS analysis showed an impact of high JAK1 expression on disease recurrence. In the case of CRC, high expression of JAK1 is associated with reduced post-progression patient survival (Hazards Ratio, HR (high) 4.28 (1.02–17.99); log rank *p* = 0.03) when compared with low JAK1 expressing patients (Figure 1C). Similarly, relapse-free survival analysis showed that high JAK1 expression is correlated with shorter recurrence-free patient survival (HR = 1.42 (1.1–1.83); log rank *p* = 0.0071) when compared with low JAK1 expressing patients (Figure 1D). Additionally, overall survival analysis of patients with high JAK1 expression supported these data by showing a trend, suggesting that shorter overall survival is correlated with high JAK1 expression (HR = 1.57 (0.96–2.56); log rank *p* = 0.071) (Figure 1E). These data support the hypothesis that increased expression of JAK1 favors CRC progression and that JAK1 expression is an important factor affecting the survival of patients with CRC. This encouraged us to focus our research objective on the potential translational regulation of JAK1 by miR-494-5p.

### 3.2. Endogenous Expression of JAK1 and miR-494-5p in CRC Cell Lines

**Figure 2:**
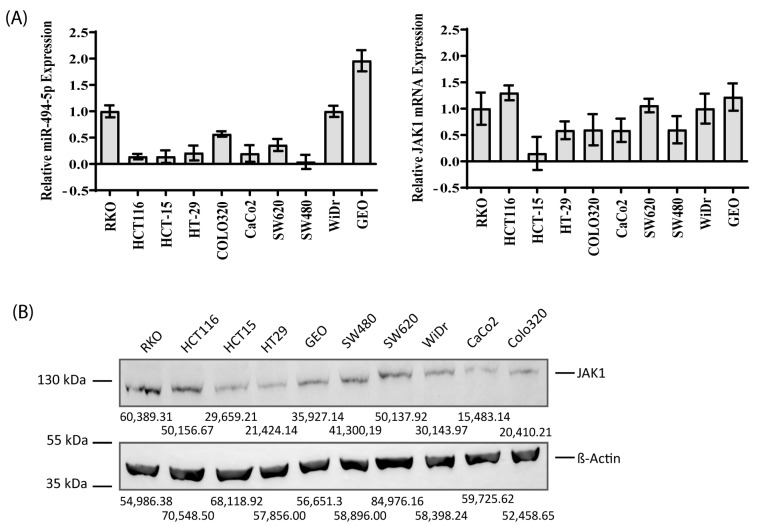
**MiR-494-5p and JAK1 expression in various CRC cell lines.** (**A**) The relative miR-494-5p and JAK1 mRNA expression in various CRC cell lines was evaluated using real-time PCR. β-Actin and RNU6 were used as housekeeping genes for normalization. The bars represent -fold change differences in their mRNA expression (±SD). (**B**) Endogenous JAK1 protein expression in various CRC cell lines (Western blot) with β-Actin as loading control. Densitometric values of each band are indicated.

To select the appropriate cell line models for our study, we determined the endogenous RNA expression of JAK1 and miR-494-5p in 10 different CRC cell lines (Figure 2A). Additionally, the endogenous protein amounts of JAK1 in all 10 cell lines were evaluated, as shown in Figure 2B. From this analysis, we have chosen RKO, HCT116, HCT15, and CaCo2 as models for our further experimental approaches.

### 3.3. miR-494-5p Represses JAK1 Expression In Vitro

**Figure 3:**
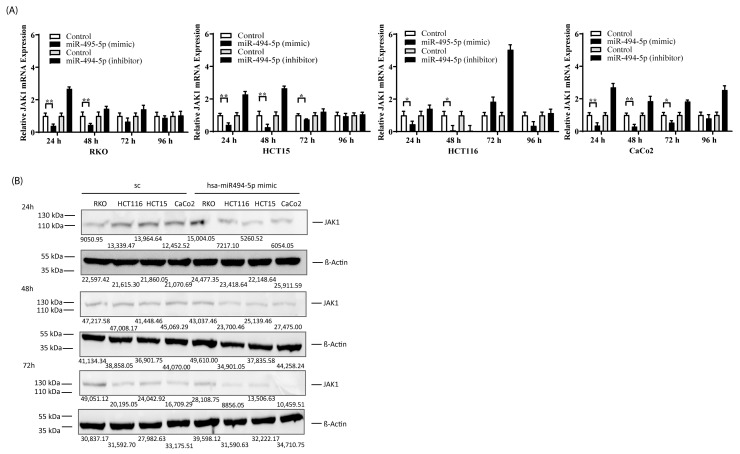
miR-494-5p represses endogenous JAK1 mRNA/protein expression in vitro. (**A**) Evaluation of JAK1 mRNA expression after transfection with miR-494-5p or a specifically designed double-stranded inhibitor, respectively (* *p* ≤ 0.05, ** *p* ≤ 0.01). (**B**) Evaluation of JAK1 protein expression after transfection with miR-494-5p. Total JAK1 and ß-Actin protein was measured 24, 48, and 72 h post-transfection. Densitometric values of each band are indicated.

Next, we determined whether the transfection of cell lines with miR-494-5p affects JAK1 mRNA and protein expression. Towards this end, RKO, HCT116, HCT15, and CaCo2 cell lines were transfected with miR-494-5p, and mRNA and protein expression of JAK1 were measured. The real-time PCR data showed that all of the cell lines transfected with miR-494-5p had a reduced amount of JAK1 mRNA, which was abrogated in the presence of a specifically designed double-stranded RNA that inhibits endogenous miR-494-5p (Figure 3A). In parallel, we observed a downregulation of JAK1 protein expression in all cell lines tested after miR-494-5p overexpression (Figure 3B). Together, this data demonstrated that miR-494-5p acts as a translational repressor in these cell lines by downregulating JAK1 mRNA and protein.

### 3.4. JAK1-3′UTR Is a Direct Physical Target of miR-494-5p

**Figure 4:**
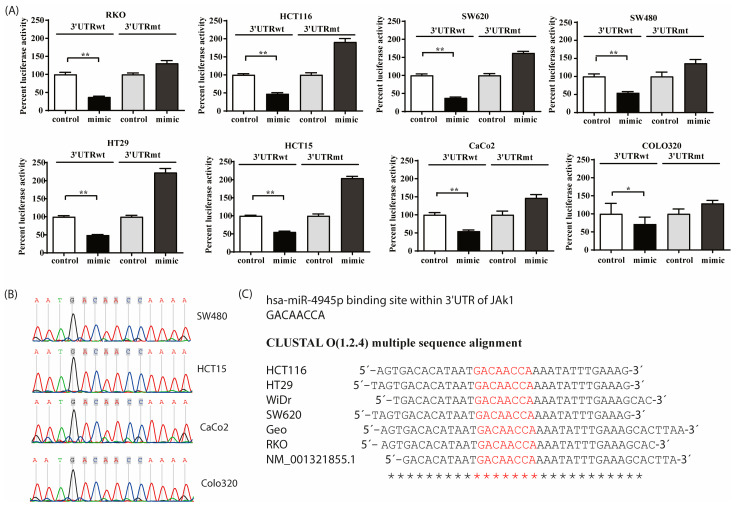
JAK1 as a direct target of miR-494-5p. (**A**) miR-494-5p targets the JAK1 3′-UTR via a functional seed sequence. Luciferase assays were conducted using various cell lines. Cells were transfected with miR-494-5p mimic and plasmids containing wild-type or mutated binding sites for miR-494-5p alongside their respective controls. The bars represent the percentage of luciferase activity (±SD) (Student’s *t*-test * *p* ≤ 0.05, ** *p* ≤ 0.01). (**B**) Genomic status of the miR-494-5p binding site in different CRC cell lines. Genomic DNA from various cell lines was PCR amplified using the primers flanking the binding site of miR-494-5p of *JAK1* 3′ UTR. The absence of any mutation within the miR binding site was verified using Sanger sequencing. (**B**) Representative sequencing chromatograph. (**C**) Sequence alignment created by CLUSTALW.

To evaluate if miR-494-5p physically binds to the putative seed sequence of JAK1 mRNA, we cloned the 3′UTR of JAK1 using primers flanking the binding site for miR-494-5p into the pLightSwitch plasmid vector downstream of the firefly luciferase reporter gene. We observed that, consistently, all cell lines transfected with miR-494-5p and the 3′UTR wild-type construct showed reduction (** *p* ≤ 0.01, except COLO320 * *p* ≤ 0.05) in reporter gene activity when compared with the respective control group (Figure 4A). Vice versa, co-transfection of miR-494-5p and a plasmid with a mutated binding site for miR-494-5p abrogated the reporter gene activity as compared to the empty vector plasmid. Furthermore, the genomic DNA from all 10 CRC cell lines was used to amplify the JAK1 3′UTR and continued to be used for Sanger sequencing. With this control, the absence of any genetic changes within the putative miR binding site was verified in all cell lines (Figure 4B,C). Together with the translational repression of endogenous JAK1 protein, as demonstrated above, these data support our conclusion that the 3′UTR of JAK1 contains a physically active functional target site for miR-494-5p in CRC cells, which is responsible for the observed translational repression of JAK1.

### 3.5. MiR-494-5p Reduces CRC Cell Proliferation

**Figure 5:**
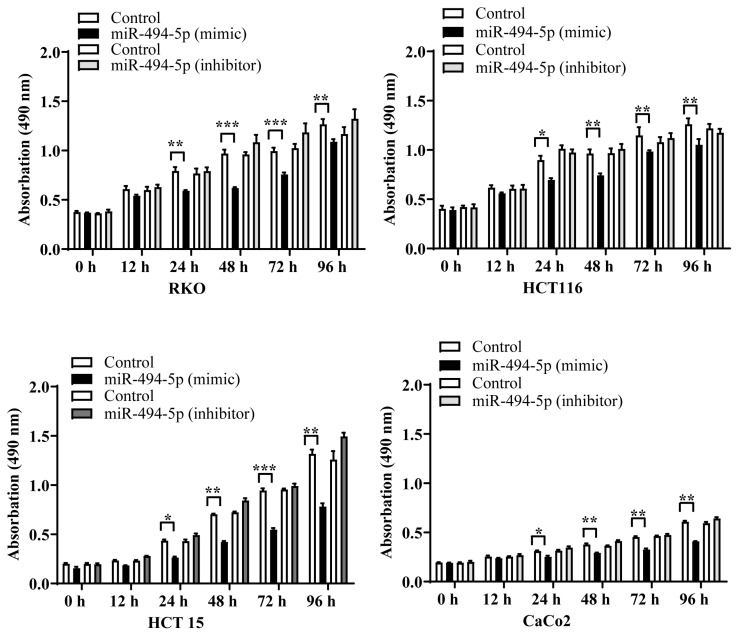
Effect of miR-494-5p on cell proliferation. MiR-494-5p reduces cell proliferation highly significantly after 48 h, which was opposed in the presence of a specific double-stranded RNA molecule that inhibits endogenous miR-494-5p. A graph of time after transfection versus absorbance at 490 nm is shown (±SD) (* *p* ≤ 0.05, ** *p* ≤ 0.01, and *** *p* ≤ 0.001).

Next, we determined if transfection of RKO, HCT116, HCT15, and CaCo2 cells with miR-494-5p affects tumor cell proliferation. We observed a reduction in the proliferation of all CRC cell lines starting after 24 h, which remained consistent until 96 h after transfection (* *p* ≤ 0.05, ** *p* ≤ 0.01, and *** *p* ≤ 0.001) (Figure 5A). This effect was abolished by co-transfection with a specifically designed double-stranded RNA that inhibits endogenous miR-494-5p. Together, these data suggest that miR-494-5p suppresses the growth of CRC cells.

### 3.6. MiR-494-5p Inhibits Constitutive Migration and Invasion of CRC Cells

**Figure 6:**
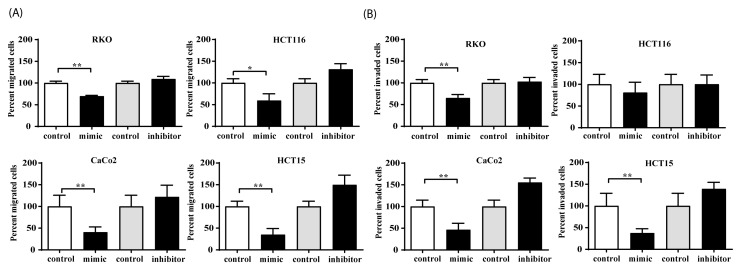
Effect of miR-494-5p on constitutive migration and invasion in vitro. (**A**) MiR-494-5p overexpression reduces migration and (**B**) invasion. Cells were transfected with miR-494-5p mimic or a specific predesigned double-stranded RNA that inhibits endogenous miR-494-5p alongside their respective controls. The complete medium of the respective cell line with 10% FBS was used as a chemoattractant. Cells were measured, and a graph of the total percent of migrated or invaded cells was plotted in each group (±S.D.) (* *p* ≤ 0.05, ** *p* ≤ 0.01).

As final functional experiments, we sought to investigate if miR-494-5p specifically regulates the initial steps of metastasis, which are migration and invasion. Therefore, RKO, HCT116, HCT15, and CaCo2 were transfected either with miR-494-5p or an inhibitory double-stranded RNA against miR-494-5p. For all cell lines, we observed a decrease in cell migration in the case of miR mimic treatment compared to the negative control groups (* *p* ≤ 0.05, ** *p* ≤ 0.01). In addition, transfection with the specific inhibitor rescued this effect, suggesting that miR-494-5p is a pivotal player in migration (Figure 6A). Similarly, all cell lines investigated showed a reduction in their invasive capability after miR transfection (* *p* ≤ 0.05, ** *p* ≤ 0.01) when compared with the negative control, which was opposed in the presence of the specific inhibitor (Figure 6B). These observations clearly emphasize that miR-494-5p is able to suppress the constitutive migration and invasion potential of CRC cells.

### 3.7. MiR-494-5p Inhibits IL-4 Stimulated JAK1, STAT6 and AKT Activation and IL-4 Induced Migration/Invasion

**Figure 7:**
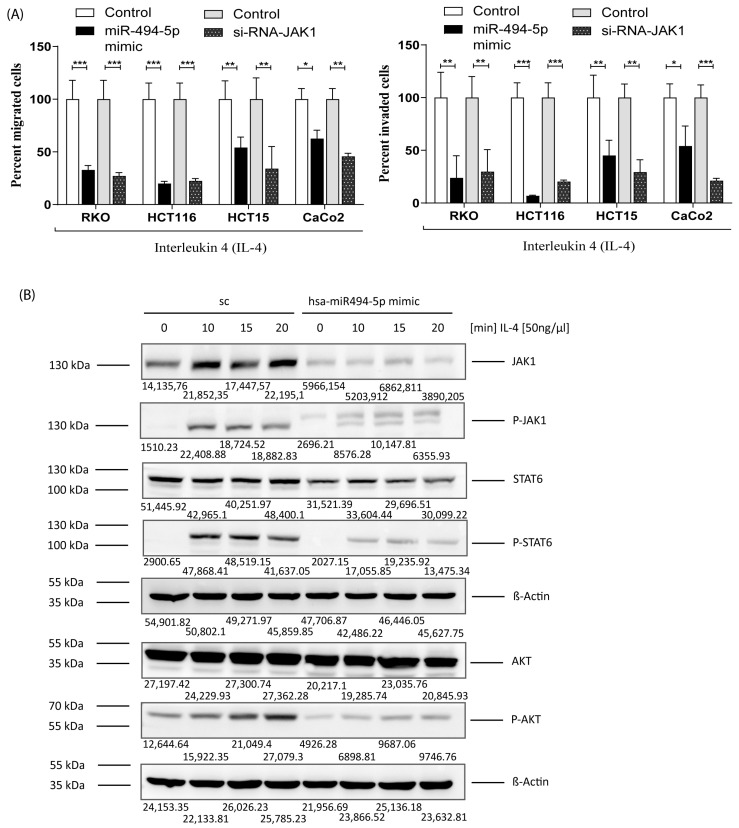
MiR-494-5p overexpression suppresses migration, invasion, and JAK1/STAT6 signaling after IL-4 stimulation. (**A**) Migration assay (**left** panel) and invasion assay (**right** panel) were conducted on RKO, HCT116, HCT15, and CaCo2 cells using miR-494-5p mimic and a si-RNA specific for JAK1, respectively, alongside their respective control. IL-4 was used as a chemoattractant. A graph of the percent of migrated or invaded cells was plotted in each group by considering all values (±S.D.) (* *p* ≤ 0.05, ** *p* ≤ 0.01, and *** *p* ≤ 0.001). (**B**) Western blot analysis demonstrating JAK1/STAT6/AKT total protein and phosphorylated status. The HCT15 cell line was used after overexpression of miR-mimic and scrambled control (sc). The cells were stimulated with IL-4 in a time-dependent manner, as depicted in the figure. The total protein amount and phosphorylation status of each protein were evaluated, and β-Actin was used as loading control. Densitometric values of each band are indicated.

IL-4 is a pleiotropic cytokine that regulates the growth, differentiation, and survival of a wide variety of cell types, including CRC. It is well established that IL-4 stimulates JAK1 phosphorylation, leading to the activation of downstream proteins, especially STAT6 or AKT, with this being a major pro-oncogenic pathway in CRC [43,45,46,47]. To answer the question of whether IL-4-mediated migration and invasion of CRC cells can be suppressed by miR-494-5p, we transfected RKO, HCT116, HCT15, and CaCo2 cells with miR-494-5p and used IL-4 as a chemoattractant. Additionally, we transfected the same cell lines with an siRNA against JAK1 to demonstrate that downregulated migration/invasion is indeed, at least to a relevant part, mediated by JAK1. In conformity with our previous results, miR-494-5p and the specific siRNA against JAK1 were able to inhibit the migration and invasion of these cell lines (* *p* ≤ 0.05, ** *p* ≤ 0.01, and *** *p* ≤ 0.001) (Figure 7A). To directly determine if STAT6 and AKT activation are regulated by the translational repression of JAK1 protein expression through miR-494-5p, HCT15 cells were transfected, as an example, with miR-494-5p mimic and stimulated with IL-4 at different time points, and the phosphorylation status of JAK1, STAT6, and AKT was determined (Figure 7B). We were able to show that stimulation with IL-4 clearly activated JAK1, STAT6, and AKT phosphorylation within a period of 10 min and that this was reduced by the translational repression brought about by miR-494-5p.

## 4. Discussion

There have been different reports demonstrating a molecular function of miR-494 in various cancers, either as a tumor suppressor miR or as an oncomiR. This ambivalent role of miR-494 and the fact that only one study so far has investigated this miR in colorectal cancer, indicating that an overexpression of miR-494 enhanced 5-FU chemosensitivity by negatively regulating DPYD [48], was our impetus to perform this investigation, based on our own previous miR expression data in CRC tumors and metastases [8].

With the present investigation, we support an interesting inhibitory role of miR-494-5p in CRC. Our experimental evidence demonstrated that an overexpression of miR-494-5p in a panel of CRC cell lines led to reduced proliferation, cell migration, and invasion, thereby supporting its role as a tumor suppressor and inhibitor of the initial steps of metastasis. We found that JAK1/STAT6 and AKT phosphorylation were reduced as a consequence of the translational repression of JAK1 through miR-494-5p. In line with our observation, several studies have demonstrated that STAT6 is highly expressed in several types of cancer and that CRC cells especially exhibit a high STAT6 activity, which is associated with increased invasion, tumor growth, and metastasis. Furthermore, high STAT6 expression is associated with lower survival rates and lymph node metastasis [40,49,50]. Similarly, an amplification of *AKT* genes has been shown in cases of ovarian [51], pancreas [52], lung [53], breast [54], melanoma [55], gastric cancer [56], and CRC [57], leading to high AKT expression. It has also been established that even in the absence of *AKT* gene amplifications, individual isoforms of AKT are aberrantly overexpressed in breast cancer, CRC [57], or hepatocellular carcinoma [58], leading to cancer progression. These data underscore our observation that an overexpression of miR-494-5p can act tumor-cell suppressive in colorectal cancer, inhibiting proliferation and initial steps of metastasis, not only through blocking the STAT6 and AKT signaling axes but specifically via targeting JAK1 protein expression.

Considering the line of evidence in the previous paragraph, it is conceivable that a low JAK1 expression and/or activity brought about by miR-494-5p inhibition led to a decrease in STAT6 and AKT expression in our present data. JAK1, as a direct target of miR-494-5p by itself, plays a pivotal role in the context of carcinogenesis and in the interaction with the immune system as suggested by our stimulation experiments with IL-4, which has been shown to be secreted by, e.g., a subset of activated T cells, mast cells, basophils, eosinophils, and neutrophils [59]. Depending on the tumor type, these types of immune cells can be found in variable proportions or the tumor microenvironment (TME) and represent different phenotypes with either pro- or anti-inflammatory properties [60]. The TME offers special microconditions able to, for example, create an immunosuppressive environment that contains, amongst others, regulatory T cells and various CD11b+ myeloid cells, including MDSC and TAMs, thereby promoting tumor growth and metastatic dissemination [61,62,63,64]. In particular, it was demonstrated that Tfh cells are a major source of IL-4 within the TMR [65]. This is of special importance since increased levels of IL-4 are commonly found in various types of primary and metastatic cancers, including CRC, and since IL-4 is known to act as a tumor-promoting cytokine [65,66,67,68,69,70,71].

Additionally, our own database analysis and several published reports have demonstrated high *JAK1* amounts and activity in tumors, rendering JAK1 as a promising biomarker that correlates with clinicopathological characteristics within specific cancer types, for example, in breast cancer [72]. Similarly, as demonstrated by Liu and colleagues, enhanced JAK1 phosphorylation was associated with poor prognosis in NSCLC [73]. Due to these reasons, JAK1 alone, its upstream regulators such as miR-494-5p, and/or its downstream target(s) are good candidates for new therapy options. Consequently, some drugs that at least target the JAK/STAT pathway are in clinical trials, exploring these possibilities in diverse cancer types, including CRC [35,74]. Particularly the JAK1/2 inhibitor ruxolitinib was shown to decrease the JAK1/2-STAT1-Mcl-1 protein level and effectively suppressed CRC cell proliferation in vitro and in vivo [75]. However, treatment of CRC often fails due to acquired chemotherapy resistance, which is one of the main reasons for an individually poor prognosis in CRC patients. Because of this, intensive work has been carried out to identify mechanisms of CRC chemoresistance, including first analyses of the role of miRs in the chemoresistance of CRC cells and on the potential use of these RNAs for CRC treatment [76,77,78]. Interestingly, various studies have already shown that the miR examined in our study can re-sensitize tumor cells to certain chemotherapeutic agents, offering one opportunity for future therapeutic strategies based on our present data in CRC. Towards this end, miR-494 has already been shown to increase 5-FU drug sensitivity when overexpressed in CRC cells [48]. Similarly, another study observed that ectopic expression of miR-494 was able to sensitize pancreatic cancer cells to 5-FU and gemcitabine [28]. Further evidence to support the role of miR-494 in overcoming chemoresistance came from Peng and colleagues, who investigated acquired drug resistance of gastric cancer cells and demonstrated an impact of miR-494 in the recovery of chemoresistance against doxorubicin [79]. Moreover, Chang and colleagues showed that the depletion of miR-494 by a circular RNA contributes to cisplatin resistance [80]. Another possible aspect for future therapeutic considerations regarding miR-494 could be its use within combinatorial therapy strategies that can target more than one pathway of cancer cells; here, various kinase inhibitors, such as imatinib, sorafinib, trastuzumab, or osimertinib, already considered for dual therapy in CRC, would be interesting partners [81,82,83,84]. Towards this end, it has already been shown that miR-494 could play a critical role during imatinib treatment in resistant prostate cancer and CML leukemic stem cells [85,86]. Additionally, high expression of miR-494 was shown to be associated with the induction of sorafinib resistance in hepatocellular carcinoma [87,88,89]. Another study aiming to explore the ability of miR-494 and FGFR2 to regulate the cancer-initiating cell phenotype and the therapeutic efficacy of lapatinib in HER2-positive gastric cancer found that increased miR-494 expression was able to reduce lapatinib resistance in these cells [90]. Additionally, the potential of miR-494 and its target genes as predictive biomarkers for breast cancer resistance to trastuzumab has already been identified by integrative bioinformatic analysis [91]. Finally, further evidence to support miR-494 as a potential future therapeutic agent came from a study that investigated mechanisms leading to resistance towards osimertinib in SCLC; here, the authors found that an elevated expression of this miR was associated with resistance to this EGFR inhibitor. Together, these data indicate that miR-494 is interesting to be considered for the development of future mono- or multi-target approaches for the personalized treatment of cancer. However, due to potentially, and at least in part, opposing roles of particular miR-494 isoforms, e.g., regarding pro- or anti-apoptosis as indicated by Alanazi et al. [92] (see below), we think that pre-clinical studies on the way to developing miRs like miR-494 as therapeutic agents still need to carefully investigate and consider specific functions and molecular actions of particular miR-494 isoforms (see below), as performed in our present study for miR-494-5p.

Nevertheless, tumor suppressive functions of the miR-494 group have been further supported by data from studies using types of cancer other than CRC. It has already been shown that at least miR-494, though not differentiating yet for particular isoforms, is generally downregulated in cholangiocarcinoma, where its overexpression induces G1/S arrest. Furthermore, xenograft experiments carried out by the same group revealed a reduction in tumor growth in vivo [93]. Additionally, Zhao and colleagues demonstrated that miR-494 is significantly lower in samples of gastric cancer patients and that overexpression of this miR inhibited in vitro proliferation, migration, and invasion in gastric cancer cells [25]. Further evidence to support a tumor suppressor role of this miR came from Yuan and colleagues. They showed that the relative mRNA expression of miR-494 in epithelial ovarian cancer tissues was downregulated when compared with the matched adjacent normal cancer tissues, its overexpression in cell lines inhibiting migration and cellular proliferation [26]. Moreover, Han and colleagues showed that miR-494 was significantly downregulated in ovarian cancer and that an overexpression of this miR resulted in reduced cell proliferation, migration, and invasion [27]. In a further study on pancreatic cancer (PC), the overexpression of miR-494-5p resulted in a significant inhibition of cell proliferation and invasion [28]. Similarly, Yang and colleagues observed low expression of miR-494 in PC and showed that miR overexpression leads to an inhibition of migration, invasion, and tumor growth [30]. In gastrointestinal stromal tumors, induced miR-494 overexpression inhibited cell proliferation, and miR-494 treatment encouraged both cell-cycle arrest and apoptosis [94]. Marino and colleagues have published data that supports the role of miR-494-5p as a tumor suppressor using miR profiling conducted on breast cancer tissues [23]. Moreover, several other groups have shown that this miR acts as a tumor suppressor in human breast cancer tissues [24,95,96] or osteosarcoma [32,97].

Finally, a study conducted by Alanazi et al. revealed miR-494 as an important part of a regulatory network that controls EGF induced apoptosis [92]. This interesting analysis after EGFR-stimulation of one single skin cancer cell line (A431) derived from an epidermoid carcinoma (not CRC) indicated that the miR-494 group negatively correlated in expression with the upregulation of pro- and anti-apoptotic genes, some of them feeding into the JAK/STAT pathway. Although direct physical targeting of JAK was not investigated in this paper and specific isoforms like miR-494-5p were not considered separately, this likely being an explanation for an association with pro- as well as anti-apoptotic genes, the authors put forward the hypothesis that the miR-494 group might regulate, or be regulated by the JAK/STAT pathway, a hypothesis which is now confirmed and specified in detail with our present paper showing JAK as the physical target of miR-494-5p. Interestingly, the same paper had implicated a (direct or indirect) targeting of PLAUR, the urokinase-receptor or u-PAR, by miR-494, an in silico finding which should be studied more intensely in the future since we and others have implicated the u-PAR and its molecular associates as one of the fundamental molecular systems to promote metastasis in almost all solid cancer entities [98,99,100]. Physical confirmation of this axis would support the development of miR-494-5p as an interesting therapeutic option (see above).

There are also data on miR-494 in other tumor types, which suggested functions opposing tumor suppressor roles, supporting the importance of considering miR-isoforms in studies and therapeutic considerations. For example, Zhang and colleagues demonstrated that miR-494 acts as a tumor promoter in NSCLC, whereby it increases cellular proliferation and colony formation [101]. Similarly, in hepatocellular carcinoma tumor tissues, endogenous miR-494 expression was higher than the expression in corresponding para-tumorous and non-tumorous tissues, and overexpression of this miR promoted cell proliferation or migration [33]. In prostate cancer, serum levels of miR-494-5p evaluated in 90 patients showed a positive correlation with tumor size and stage. This study also proposed that circulating miR-494 in the serum could be used as a potential biomarker for the diagnosis of prostate cancer [34].

Reasons explaining these discrepancies in the observed functions of miR-494 can be severalfold. One possible most important explanation, in our opinion, is based on the discovery of isomiRs resulting from RNA modifications mediated by, e.g., deaminases and exonucleases, the resulting isoforms differing from the canonical ones in length, sequence, or both. In consequence, these variations of mature miRs can be responsible for distinct or divergent functions compared to their related canonical counterparts, a notion that needs to be taken into account most critically when particular isoforms might be chosen for biomarker or drug development (see above) [102]. Moreover, it was demonstrated that isomiR expression varies among different tissues and cancer types, depending on the conditions of the cancer cell or tissue context, and affects different molecular targets. Thus, differentially expressed isomiRs could be used to discriminate between normal and cancer tissues and different tumor types [103]. Consequently, such isomiRs can initiate different, in part opposing, functional changes in the cancer cells that could extend or change the canonical microRNA’s role by acquiring or losing different targets, as it was demonstrated, for example, with miR-140-3p and its 5′isomiR. Both versions are upregulated in breast cancer tissue, but besides their tumor-suppressive role, they affect different pathways [104]. Many other isomiRs and their canonical counterparts exhibit different functions, such as miR-411 and its 5′isomiR, whereby the expression of isomiR-411 rapidly decreases while the canonical miR-411 is increasing, in response to acute ischemia [105]. A transcriptional repression of TGFB is achieved by canonical miR-411, resulting in a pro-angiogenic phenotype, while the 5′isomiR is responsible for decreased cell migration and a suppression of angiogenesis by transcriptional repression of F3 and ANGPT1 [105].

It is also important to note that one miR has the potential to target the expression of multiple target mRNAs, while, vice versa, each mRNA may be targeted by multiple miRs [106]. In consequence, this complex regulation of different molecular targets could additionally explain the different, and in part opposing, functional changes in the investigated specific cancer models. Nevertheless, most recent reports dealing with miRs like miR-494 describe only one-to-one relationships between this miRNA and their individual target [25,26,107,108,109,110,111]. Certainly, our own present in vitro study can depict a small part of this regulatory network only. Larger future network studies, including groups of miRs or possible isomiRs, their targets, and specific targeting conditions in particular scenarios and tumor types, could answer this.

Nevertheless, taken together, our present study suggests that JAK1 is negatively regulated at the post-transcriptional level by miR-494-5p via a specific, physically active target and seed sequence motif within the JAK1-3′UTR. Furthermore, miR-494-5p reduces proliferation, migration, and invasion in CRC cells. Together with the impact on IL4-signalling via the JAK1/STAT6 axis, our data strongly support an early tumor suppressive role of miR-494-5p in CRC. Consequently, our data suggest miR-494-5p as a suppressor of early steps of CRC carcinogenesis and metastasis, which might be of interest for future biomarker studies or even treatment strategies.

## 5. Conclusions

In conclusion, we found that an overexpression of miR-494-5p leads to a significant reduction in JAK1 mRNA and protein expression in CRC cells and that the JAK1-3′UTR is a physical target of miR-494-5p in CRC. Moreover, cell proliferation, migration, and invasion are significantly reduced after miR-494-5p overexpression. Finally, an overexpression of this miR leads to a reduced JAK1, STAT6, and AKT phosphorylation and IL-4-induced migration and invasion; this, together with data from the literature, leads to the conclusion that miR-494-5p is an early tumor and initial metastasis suppressor in CRC.

## Data Availability

Data are contained within the article or supplementary material.

## Supplementary Materials

The following supporting information can be downloaded at https://www.mdpi.com/article/10.3390/cancers16010024/s1, Figure S1: Uncropped blots and densitometry values for Figure 2B; Figure S2; Uncropped blots and densitometry values for Figure 3B; Figure S3: Uncropped blots and densitometry values for Figure 3B; Figure S4: Uncropped blots and densitometry values for Figure 7B; Figure S5: Uncropped blots and densitometry values for Figure 7B; Table S1: Unranked list of all 169 potential targets of miR-494-5p found by merging the results of the search tools TargetScanHuman_8.0, miRDB, and miRWalk, in alphabetical order.

## Abbreviations

3′ untranslated region (3′UTR); actin beta (ACTB); American type culture collection (ATCC); angiopoetin 1 (ANGPT1); bovine serum albumin (BSA); chronic myeloid leukemia (CMC); colorectal cancer (CRC); complementary DNA (cDNA); database for annotation, visualization and integrated discovery (DAVID); deoxyribonucleic acid (DNA); gene expression profiling interactive analysis (GEPIA); coagulin factor III (F3); European genome-phenome archive (EGA); epidermal growth factor receptor (EGFR); fibroblast growth factor receptor 2 (FGFR2); gene expression omnibus (GEO); homo sapiens (hsa); human epidermal growth factor receptor (HER) 2; integrin subunit alpha M (CD11b); interleukin 4 (IL-4); miRNA isoforms (isomiRs); janus kinase (JAK); Kyoto encyclopedia of genes and genomes (KEGG); microRNAs (miRs); myeloid-derived suppressor cells (MDSC); overall survival (OS); post-progression survival (PPS); phosphate-buffered saline (PBS); protein kinase B (PKB), (also known as AKT); ribonucleic acid (RNA); RNA U6 small nuclear 2 (RNU6-2); relapse free survival (RFS); small cell lung cancer (SCLC); signal transducers and activators of transcription (STAT); sodium dodecyl sulfate–polyacrylamide gel electrophoresis (SDS-PAGE); T follicular helper cells (Tfh); tumor-associated macrophages (TAM); the cancer genome atlas (TCGA); the genotype-tissue expression (GTEx); tumor microenvironment (TME); polymerase chain reaction (PCR); tris-buffered saline (TBS); TBS with Tween (TBST); transforming growth factor beta (TGFB).
