# Supplementary material for: JAK1 Is a Novel Target of Tumor- and Invasion-Suppressive microRNA 494-5p in Colorectal Cancer
Source: Cancers (Basel). 2023 Dec 20;16(1):24. doi: 10.3390/cancers16010024 (PMC10778350; doi:10.3390/cancers16010024)
Supplement: Supplementary file 1 [file cancers-16-00024-s001.zip › cancers-2767680-supplementary.pdf]

Supplementary Material: JAK1 is a novel target of tumor- and invasion-suppressive microRNA 494-5p in colorectal cancer

Supplementary Table S1: Unranked list of all 169 potential targets of miR-494-5p found by merging the results of the search tools TargetScanHuman_8.0, miRDB, and miRWalk, in alphabetical order.

Gene Symbol			
ABCC1	CSNK1A1L	KRT33B	SERPINB2
ABCG4	CYP1B1	KY	SETD5
ADAMTS15	DMRT2	LMAN1	SETD7
ADAMTS3	DNAJB5	LPAR4	SFRP5
AHR	DPH2	LRRC8A	SH3RF3
AIFM1	DPH3	MEOX2	SHANK2
ALDOB	DPP3	MPC1	SHMT1
ALG9	DTNA	MPDZ	SLC25A36
ALOX15B	DUSP13	MXD1	SLC39A9
ANKRD34C	EBLN2	MXI1	SLC41A2
ARHGAP29	EDNRA	MYO7A	SLC9A1
ARID4A	EGLN1	NDP	ST3GAL6
ARPP19	EIF2AK2	NHLH2	STK38
ARRDC3	EPHA4	NIPAL1	SVOP
ATP1B4	EPHB2	NLK	SYNGR3
ATP6V1G1	ERBB2	NPAS3	SYT9
BCL11B	FAM219B	NUFIP2	TBC1D23
BCL2	GATC	NUP210	TBC1D2B
BCL2L14	GBP1	OAS2	TET3
BET1L	GGA2	PAK2	TMCC1
BOD1L2	GORASP2	PCDH11X	TMEM115
BRPF3	GPC6	PCTP	TMEM245
BTAF1	GPR63	PEBP1	TMEM26
BTBD11	GSK3B	PIK3R3	TMTC1
C4orf19	HAPLN4	PLOD1	TNFSF10
C9orf72	HLA-DPB1	PPP1R12B	TP53RK
CABYR	HOMEZ	PRICKLE2	TRMT6
CADM2	HSD17B11	PRKCA	TRPC3
CALB1	HSPD1	PRKG1	TRPS1
CAMK2B	IGLL5	PROS1	TSPAN11
CCDC6	IL1A	PTBP2	TTC23L
CD274	IL6ST	RAD23B	TXNL1
CDC42SE1	IPMK	RANBP6	UMODL1
CDKN2AIP	ITGA6	RCOR1	USP34
CDKN2B	ITGBL1	RHOBTB1	YIPF6
CDV3	ITPR2	RNF24	ZFP82
CEBPG	JAK1	RORA	ZFYVE16
CHP1	KAT2B	RUNX1T1	ZMYM3
CLASP2	KCNJ16	SAV1	ZNF131
CLMN	KCNJ9	SCMH1	ZNF850
CORO2A	KCTD9	SCN11A	
CRMP1	KIF5C	SCUBE3	
CSNK1A1	KLHL12	SEMA6B	

Supplementary Figure S1: Uncropped blots and densitometry values for Figure 2B.

Blots relative to Figure 2B

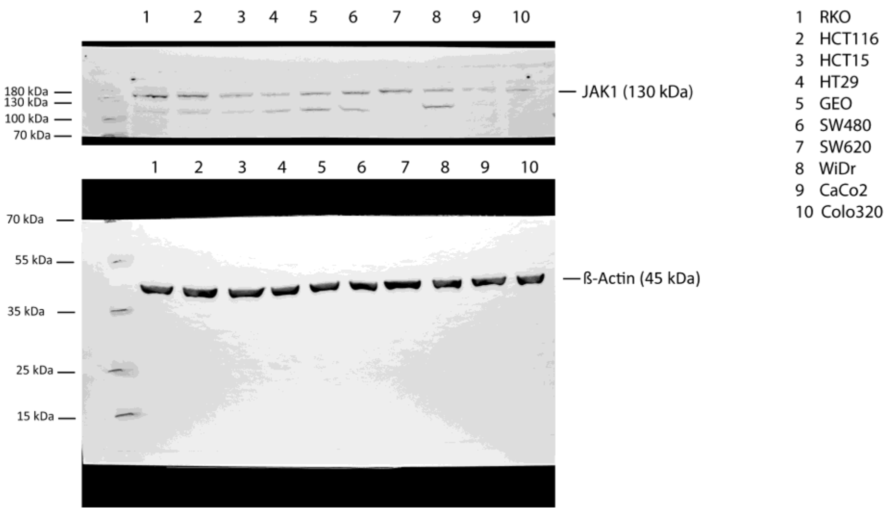

total JAK1		Actin	
1	60389,31	1	54986,38
2	50156,67	2	70548,501
3	29659,21	3	68118,915
4	21424,14	4	57855,995
5	35927,14	5	56651,359
6	41300,19	6	58895,995
7	50137,92	7	84976,158
8	30143,97	8	58398,238
9	15483,14	9	59725,622
10	20410,21	10	52458,652

Supplementary Figure S2: Uncropped blots and densitometry values for Figure 3B.

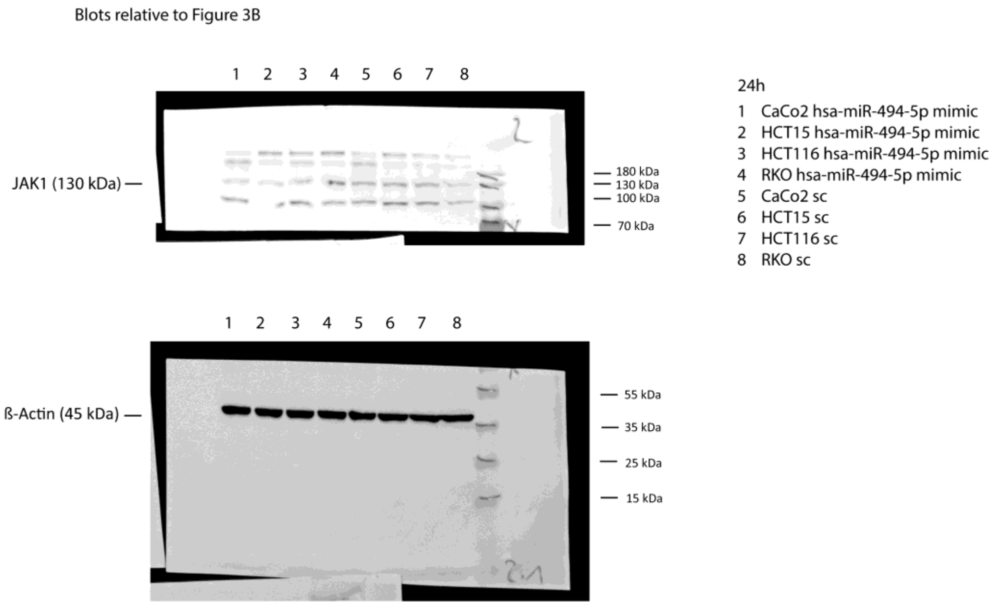

24 hours			
total JAK1		Actin	
1	6054,054	1	25911,589
2	5260,518	2	22148,64
3	7217,104	3	23418,64
4	15004,05	4	24477,347
5	12452,52	5	21070,69
6	13964,64	6	21860,054
7	13339,47	7	21615,296
8	9050,953	8	22597,418

Supplementary Figure S3: Uncropped blots and densitometry values for Figure 3B.

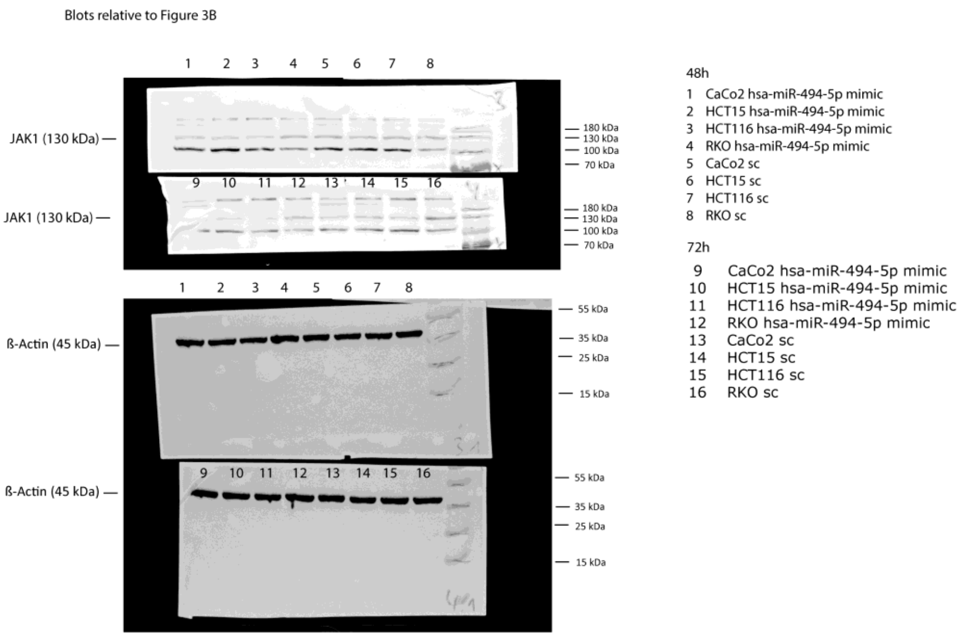

48 hours			
total JAK1		Actin	
1	27475	1	44258,238
2	25139,46	2	37835,581
3	23700,46	3	34901,045
4	43037,46	4	49609,995
5	45069,29	5	44069,995
6	41448,46	6	36901,752
7	47008,17	7	38858,045
8	47217,58	8	41134,338

72 hours			
total JAK1		Actin	
9	10459,51	9	34710,752
10	13506,63	10	32222,167
11	8856,045	11	31590,631
12	28108,75	12	39598,116
13	16709,29	13	33175,51
14	24042,92	14	27982,631
15	20195,05	15	31592,702
16	49051,12	16	30837,167

Supplementary Figure S4: Uncropped blots and densitometry values for Figure7B**.**

Blots relative to Figure 7B

1 HCT15 sc 0 min
2 HCT15 sc 10 min
3 HCT15 sc 15 min
4 HCT15 sc 20 min
5 HCT15 hsa-miR-494-5p mimic 0 min
6 HCT15 hsa-miR-494-5p mimic 10 min
7 HCT15 hsa-miR-494-5p mimic 15 min
8 HCT15 hsa-miR-494-5p mimic 20 min

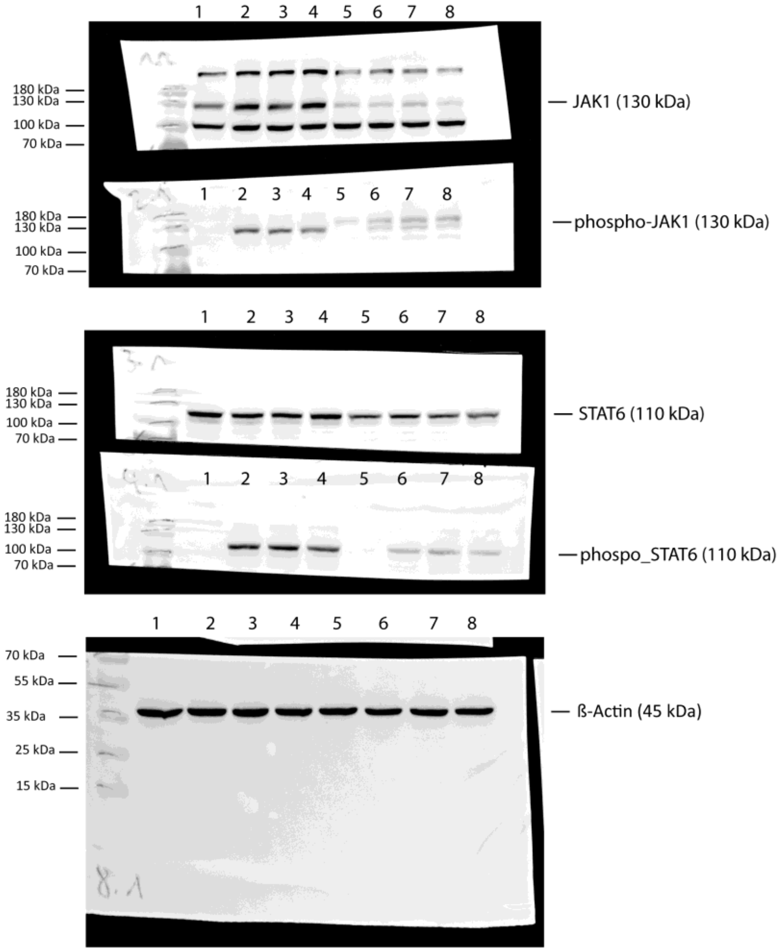

total JAK1		Phospho JAK1	
1	14135,76	1	1510,225
2	21852,35	2	22408,882
3	17447,57	3	18724,518
4	22195,1	4	18882,832
5	5966,154	5	2696,205
6	5203,912	6	8576,276
7	6862,811	7	10147,811
8	3890,205	8	6355,933

total STAT6		phospho STAT6	
1	51445,92	1	2900,652
2	42965,1	2	47868,409
3	40251,97	3	48519,146
4	48400,1	4	41637,045
5	31521,39	5	2027,146
6	33604,44	6	17055,853
7	29696,51	7	19235,924
8	30099,22	8	13475,338

Actin for JAK1/phosho JAK1 and STAT6/phospho STAT6	
1	54901,82
2	50802,1
3	49271,97
4	45859,85
5	47706,87
6	42486,22
7	46446,05
8	45627,75

Supplementary Figure S5: Uncropped blots and densitometry values for Figure7B**.**

Blots relative to Figure 7B

1 HCT15 sc 0 min
2 HCT15 sc 10 min
3 HCT15 sc 15 min
4 HCT15 sc 20 min
5 HCT15 hsa-miR-494-5p mimic 0 min
6 HCT15 hsa-miR-494-5p mimic 10 min
7 HCT15 hsa-miR-494-5p mimic 15 min
8 HCT15 hsa-miR-494-5p mimic 20 min

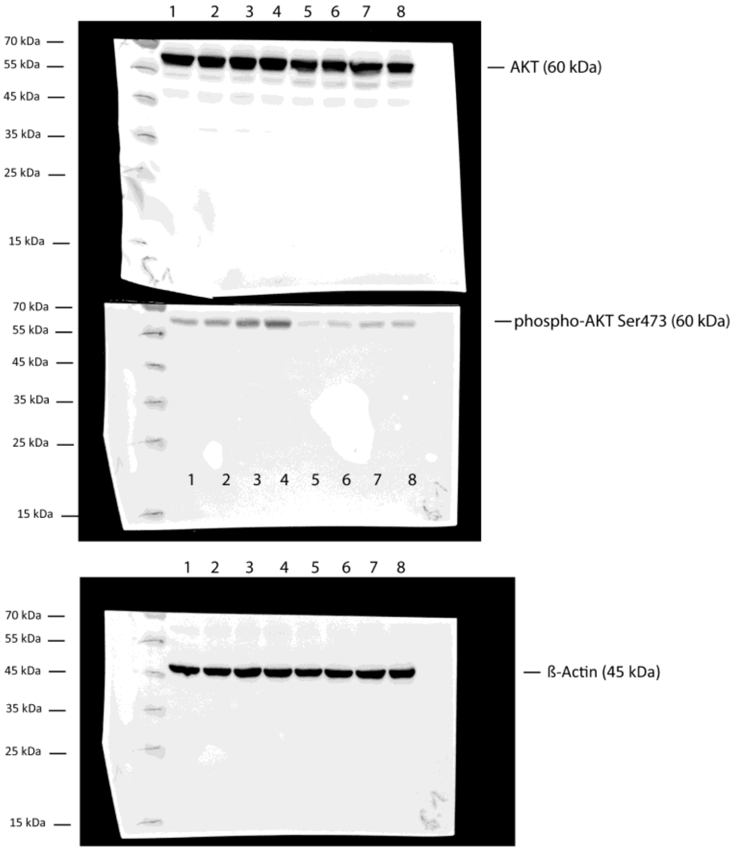

total AKT		phospho AKT		
1	27197,42		1	12644,64
2	24229,93		2	15922,35
3	27300,74		3	21049,4
4	27362,28		4	27079,3
5	20217,1		5	4926,276
6	19285,74		6	6898,811
7	23035,76		7	9687,054
8	20845,93		8	9746,761

Actin for Akt	
1	24153,35
2	22133,81
3	26026,23
4	25785,23
5	21956,69
6	23866,52
7	25136,18
8	23632,81
